# South Korea’s population shift: challenges and opportunities

**DOI:** 10.1016/j.lanwpc.2023.100865

**Published:** 2023-07-31

**Authors:** The Lancet Regional Health – Western Pacific

World Population Day, an event dedicated to raising awareness of the importance of population issues is celebrated on July 11 every year. Population issues are especially critical in South Korea. This nation, formally known as the Republic of Korea, and home to 53.4 million people, is the fourth-largest economy in Asia. Recently, the nation saw its citizens become 1 to 2 years younger on paper by passing a law whereby a person is aged 0 years at birth, instead of 1 year old based on the traditional Korean age system. This law has been passed in the context of a major demographic shift in the nation. South Korea has one of the fastest ageing populations and the lowest birth rate in the world. The country is on route to becoming a so-called super-aged society by 2025, whereby the proportion of people aged 65 years and over will reach 20% of the total population. By 2050, this proportion is anticipated to increase to as high as 44%. The way in which South Korea deals with this imminent demographic shift could define its population health prospects over the next decades.

The health status of the South Korean population is relatively good. The country, together with Japan and Singapore, consistently ranks in the top 25 healthiest nations in the world in terms of life expectancy, environmental factors, and health risks. Like other countries in the Western Pacific region, non-communicable disease contributes the most to disease burden. The top three causes of death are stroke, ischaemic heart disease, and lung cancer. Mortality due to Alzheimer’s disease increased by 74.3% between 2009 and 2019, a much greater increase than stroke (16.6%), ischaemic heart disease (33.4%), or lung cancer (40.5%) over the same period. Based on a large nationwide South Korean cohort, Yang and colleagues identified that disability, chronic diseases, and advanced age were the main risk factors for stroke. To counteract the economic and social ramifications of a super-aged society, several national strategies and policies focused on reversing the plummeting birth rate have been implemented. In 2022, the average number of children a woman would bear in her lifetime in South Korea was at an all-time low; 0.78, much lower than the Organization for Economic Cooperation and Development (OECD) average of 1.3–1.9 children per woman. The newly assembled team on population policy led by the finance and health ministries plans to review and further promote policies addressing the low birth rate and ageing population as well as population structure.

To be effective, these strategies and policies need to address the underlying sociocultural issues faced by older people, women, and youth in South Korea. Despite a higher life expectancy, South Korea has one of the highest rates of poverty among older people in the OECD. More than 40% of people aged 65 years and over face relative income poverty. That is, an income lower than 50% of the median household disposable income. In the OECD, South Korea ranked amongst one of the lowest countries with regards to working conditions for women in terms of gender pay gap, parental leave, childcare costs, educational attainment, and representation in management and leadership roles. Furthermore, some negative attitudes towards younger people (eg, no-kids zones), compounded by a high cost of living and an expensive and competitive education system, contribute to creating a challenging environment in which to raise children.

While understandably a large focus of government policy is to address the ageing population and low birth rate, strengthening primary health care as a key approach to preparing for and managing the demographic shift has perhaps been largely overlooked. Although South Korea’s universal health-care system is considered one of the best worldwide, it largely focuses on hospital care instead of primary care. For example, 90% of medical facilities are privately owned, compared with public health-care centres managed by the local government, which tend to be used more frequently by older people and people with lower incomes. Furthermore, limited financial incentives are available to provide preventive care in primary care settings. A strong primary care system can help reduce avoidable hospitalisations and health-care spending and improve health outcomes. Park and colleagues highlighted that healthy weight management interventions could potentially reduce the risk of development of female predominant cancers depending on malignancy type and menopausal status. Similarly, preconception care interventions could contribute towards improving pregnancy outcomes and reducing maternal and child mortality and morbidity.

Population and health policies must go hand in hand. Policies must shift to focus more on health promotion, preventive care, and disease management, especially for women and couples planning to start a family as well as older people, particularly those with chronic disease. A comprehensive set of primary health-care services tailored to the underlying sociocultural issues of South Korea’s demographic shift is a fruitful avenue towards the health of current and future generations of South Koreans.

